# Deep Venous Thrombosis in a 62-Year-Old Female With Schizoaffective Disorder Who Developed COVID-19

**DOI:** 10.7759/cureus.11421

**Published:** 2020-11-10

**Authors:** Maria Ruiza Yee, Adeolu O Oladunjoye, Larry Rotenberg

**Affiliations:** 1 Psychiatry, Drexel University College of Medicine, Philadelphia, USA; 2 Psychiatry, Philadelphia Collge of Osteopathic Medicine, Philadelphia, USA; 3 Psychiatry, Reading Hospital Tower Health, West Reading, USA; 4 Medical Critical Care, Boston Children's Hospital, Boston, USA

**Keywords:** dvt, covid-19, psychiatric patient

## Abstract

COVID-19 pandemic cases first started in November 2019 in Wuhan, China. However, the origins of the virus remain uncertain. Information and clinical complications of the disease, as well as treatment, continue to evolve in real-time. The latest complication of thrombolytic events of COVID-19 was thrust into the spotlight following the Associated Press report of Broadway star Nick Cordero having to undergo leg amputation for deep venous thrombosis (DVT) in April 2020 and eventually succumbing to the infection. DVT is a subset of venous thromboembolism and is a preventable cause of morbidity and mortality. Here, we describe a woman with serious mental illness developing COVID-19 and DVT complications.

## Introduction

The first cases of severe acute respiratory syndrome coronavirus 2 (SARS-CoV-2) were reported in Wuhan City, China, as pneumonia. Coronaviruses are a large family of zoonotic viruses and are transmissible between animals and humans. These viruses can cause illnesses ranging from the common cold to more severe conditions such as the Middle-East Respiratory Syndrome (MERS), and Severe Acute Respiratory Syndrome (SARS). SARS-CoV-2 cases would later be confirmed as coronavirus -19 (COVID-19) and spread like a pandemic throughout the globe. The U.S. Centers for Disease Control and Prevention (CDC) identifies the common symptoms like fever or chills, shortness of breath, and cough [1]. As of October 16, 2020, the Johns Hopkins University (JHU) estimated that over 39 million people had contracted COVID-19 worldwide [2]. Though the initial presentation for COVID-19 was respiratory, evolving cases show involvement of the heart, kidney, liver and more recently, blood clots such as DVT.

Patients with serious COVID-19 infection are more likely to have COVID-19 associated coagulopathy characterized by a cytokine storm, systemic inflammatory response and coagulopathy [3]. Elevations characterize the most common pattern of coagulopathy in hospitalized patients in fibrinogen, D-dimer levels and mild prolongation of prothrombin time/activated partial thromboplastin time (PT/aPTT). This correlates with a parallel rise in markers of inflammation (e.g. C-reactive protein). Unlike the pattern is seen in classic disseminated intravascular coagulopathy (DIC) from bacterial sepsis or trauma, prolongation of PT/aPTT (prothrombin time/activated partial thromboplastin time) is minimal, thrombocytopenia (platelet count 100 x 109/L), and lab results supporting microangiopathy are infrequent [4]. It is postulated that COVID-19-induced coagulopathy (CIC) is more prothrombotic than hemorrhagic and maybe an uncontrolled immunothrombotic response to COVID-19 [3]. Those who die from COVID-19 coagulopathy are those who meet the criteria for DIC including markedly increasing D-dimer levels (3- to 4- fold) and fibrin degradation product (FDP) over time, and longer PTT/aPTT. There is overall mortality of 11.5%, 71.4% meeting the criteria for DIC [5]. Anticoagulant therapy mainly with low molecular weight heparin appears to be associated with better prognosis [6].

## Case presentation

A 62-year-old white female has a long history of schizoaffective disorder, bipolar type, since age 50, when she was first hospitalized on an involuntary civil commitment. Since then, the patient has had 10 psychiatric hospitalizations, including hospitalization at the state hospital for two years. She has had episodes of manic psychosis and depression. She also has borderline intellectual functioning. Fortunately, she has never had any suicide attempts. She was tried on numerous psychotropic medications including different adequately dosed antipsychotic medications as well as antidepressant medications for an adequate duration to ensure that they were on the therapeutic dose and ultimately, she was stabilized on a combination of clozapine and sertraline. There was no drug or alcohol involvement. Medical history is significant for hypothyroidism, history of basal cell carcinoma status post excision, osteopenia, vitamin-D deficiency, history of simple and complex endometrial hyperplasia without atypia, status post total abdominal hysterectomy and bilateral salpingo-oophorectomy. She is allergic to penicillin. Family history is significant for alcoholism in father and possibly post-traumatic stress disorder. Mother had a “nervous breakdown”. There was a significant family history of psychosis in maternal grandmother, and drug and alcohol abuse in brothers. The patient had a distant relationship with her parents. She was raped three times. In fact, her son is the product of one of the rapes. The patient is single. She has never been married. Due to the severity of her psychiatric illness, the patient has been on government disability benefit for the past nine years.

**Figure 1:**
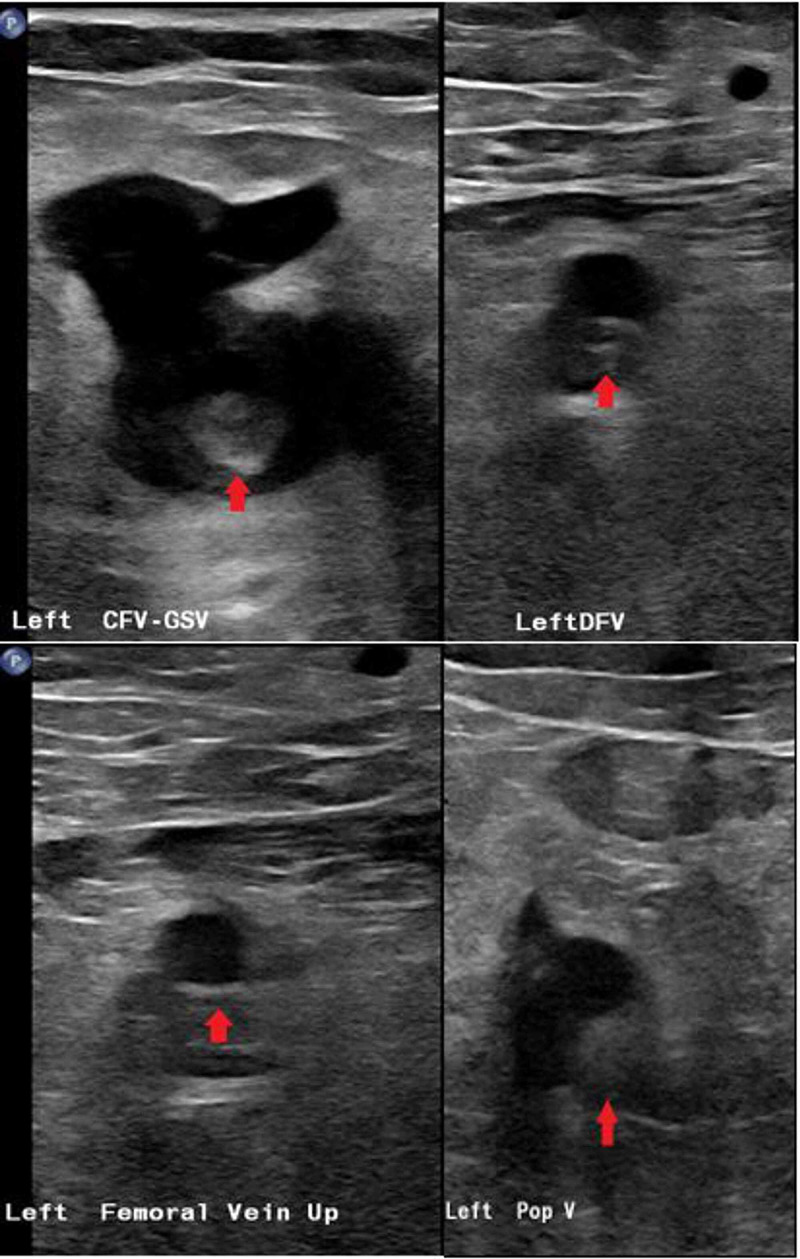
Ultrasound of the left lower extremity vein Extensive lower extremity deep venous thrombosis of the left lower extremity vein (red arrows). CFV, common femoral vein; GSV, Great saphenous vein; DFV, Deep femoral vein; Up, Upper area; Pop V, Popliteal vein

**Figure 2:**
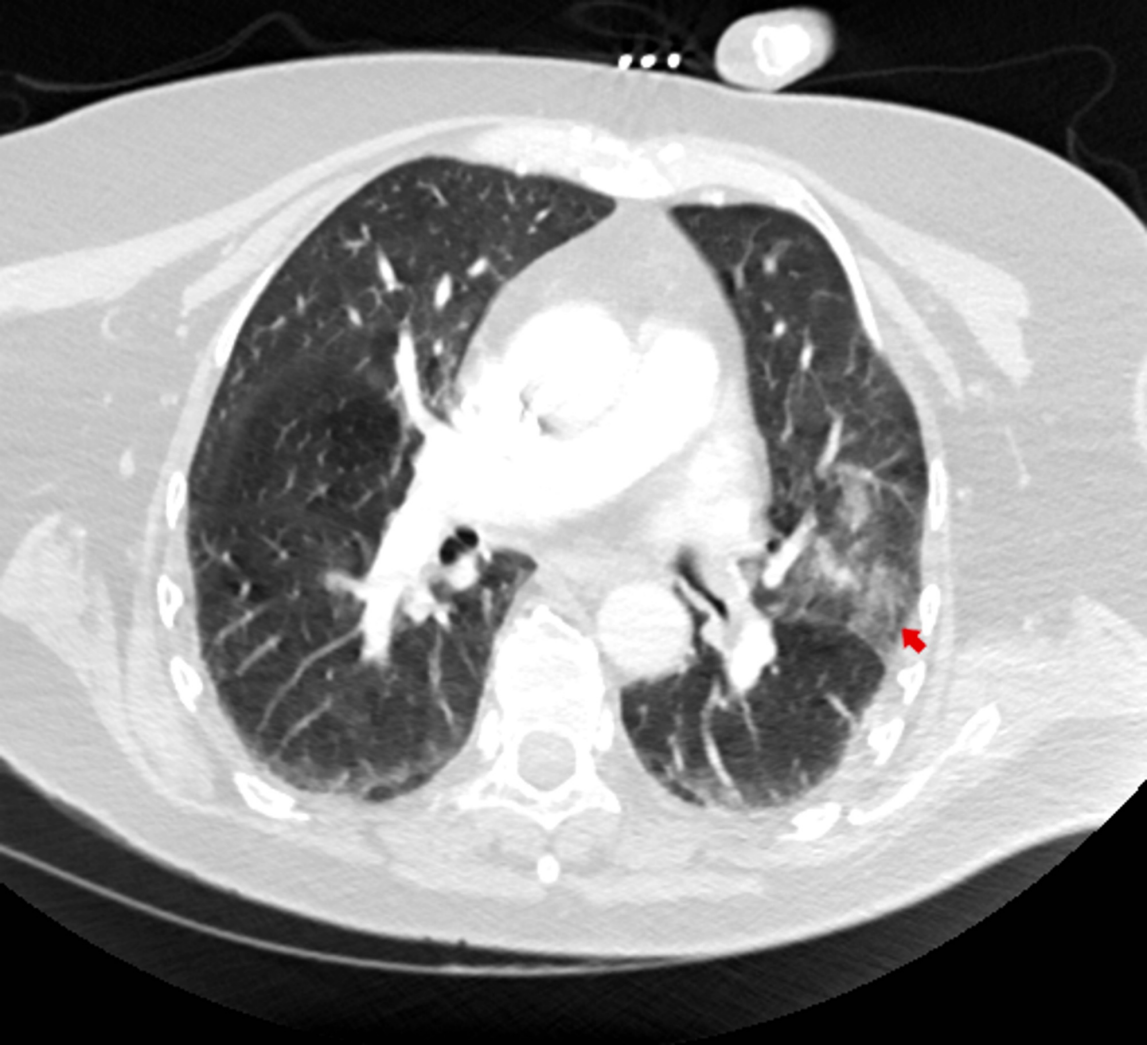
Computed Tomography Angiography for Pulmonary Embolism (CTA PE) There is no evidence of pulmonary embolism but there are ground-glass opacities compatible with left lower lobe pneumonia (red arrow)

The patient is in treatment at a clozapine clinic at a not for profit outpatient clinic of a hospital system. During one of the visits, she appeared unwell, febrile with a temperature of 99.50 F and white blood cell count of 15.3 x 109/L (reference range: 4.8-10.8 x 109/L). She was a poor historian because of her limited intellectual capacity. She denied any physical symptoms except for leg and knee pain, but with her white blood cell count being elevated at 15.3 x 109/L, she was referred to the emergency room because of clinical suspicion of some severe underlying medical conditions like pneumonia, meningitis or cardiovascular disease. While in the emergency room, her leg was noted to be swollen. A left lower extremity ultrasound revealed extensive left lower extremity (external iliac, femoral, and popliteal) DVT (Figure 1). Computed tomographic pulmonary angiography for pulmonary embolism (CTA PE) chest was negative for pulmonary embolism but did show ground-glass opacities compatible with left lower lobe pneumonia (Figure 2). She did not have any evidence of phlegmasia cerulea dolens. Her inferior vena cava was patent, and the thrombus did not extend into the common iliac vein on the left. She was subsequently admitted to the medical floor. Diagnostic laboratory tests were taken on admission and on day three (Table 1). She was started on a heparin drip. Vascular surgery was consulted, and no surgical intervention was necessary. She has eventually switched to rivaroxaban 15 mg two times a day for seven days and then 20 mg daily with dinner. Pneumonia was treated with a course of ceftriaxone and doxycycline combination which she completed. Due to the extensive DVT without any previous history nor family history of blood clots, COVID-19 was suspected. SARS-CoV-2 test was positive.

 

**Table 1:** Diagnostic Laboratory Test PT, Prothrombin time; aPTT, activated partial thromboplastin time; CRP, C-reactive protein; ref, reference

Day from admission	D-dimer (ref range: <0.50 ug/ml)	PT/aPTT (ref range:11.6-13.9/30-40 seconds)	CRP (ref range: <1mg/dL)	Platelet count (ref range: 130-400 x 10^9^/L)
Day 0	14.83 ug/ml	1.4/16.4 seconds	11.62 mg/dL	132 x 10^9^/L
Day 3	4.67 ug/ml	1.4/16.7 seconds	4.67 mg/dL	177 x 10^9^/L

## Discussion

COVID-19 remains a pandemic. Though the initially reported cases were respiratory in presentation, other organ systems have now been reported to be involved. Mortality from this infection is high [2]. Complications keep evolving as we know more about the illness. Coagulopathy now seems to be another complication of this viral infection. DVT is a major preventable cause of morbidity and mortality. Venous thromboembolism (VTE) which includes DVT and pulmonary embolism (PE) affects an estimated 1 per 1,000 people and contributes to 60,000 - 100,000 deaths annually [7]. It is postulated that COVID-19-induced coagulopathy (CIC) is more prothrombotic than hemorrhagic and maybe an uncontrolled immunothrombotic response to COVID-19 [3]. A high clinical suspicion is necessary in order to reduce mortality from coagulopathy because early treatment leads to improved prognosis as well as reduced risk of mortality [6]. Patients with severe psychiatric illnesses are not immune to the scourge of this infection, as our case supports. Moreover, in this particular situation, the patient was unaware of any exposure to COVID-19. But, due to the extensive DVT without a prior history nor a family history of blood clots, COVID-19 was suspected. Without a high level of clinical suspicion, further diagnostic workup would not have been pursued, which could have led to a poorer outcome in this patient. Fortunately, this patient did not have evidence of DIC as her platelet count was lower than would be expected in DIC, as is her PT/aPTT prolongation. Anticoagulant therapy mainly with low molecular weight heparin appears to be associated with better prognosis [6] as evidenced by this patient’s good outcome.

A high level of clinical suspicion is necessary, especially when dealing with patients with severe mental illness and cognitive dysfunction as they are poor historians and have a poor understanding of medical issues which can lead to dire consequences. Hence, it falls upon the clinician to tease out what could be going on with the patient. Especially during this COVID-19 pandemic, all patients complaining of chest pain or leg pain, not just those with pulmonary symptoms, need to be suspected of COVID-19 unless proven otherwise.

## Conclusions

Patients with serious mental illness are not immune to the scourge of COVID-19. If anything, a clinician should have a high level of clinical suspicion with these patients as they are usually poor historians and have limited understanding of medical conditions. It behoves psychiatrists to be cognizant of the possibility of COVID-19 in our patients, especially during this pandemic, including other complications such as coagulopathy. Mortality in this condition is high. Early and proper treatment leads to a reduced risk of mortality and morbidity.

## References

[1] (2020). Coronavirus Disease 2019 (COVID-19): Symptoms of Coronavirus. https://www.cdc.gov/coronavirus/2019-ncov/symptoms-testing/symptoms.html.

[2] (2020). COVID-19 Dashboard by the Center for Systems Science and Engineering (CSSE) at Johns Hopkins University (JHU). https://coronavirus.jhu.edu/map.html.

[3] Barnes G, Burnett A, Allen A (2020). Thromboembolism and anticoagulant therapy during the COVID-19 pandemic: interim clinical guidance from the anticoagulation forum. J Thromb Thrombolysis.

[4] (2020). COVID-19 and Coagulopathy: Frequently Asked Questions. https://www.hematology.org/covid-19/covid-19-and-coagulopathy.

[5] Tang N, Li D, Wang X, Sun Z (2020). Abnormal coagulation parameters are associated with poor prognosis in patients with novel coronavirus pneumonia. J Thromb Haemost.

[6] Tang N, Bai H, Chen X, Gong J, Li D, Sun Z (2020). Anticoagulant treatment is associated with decreased mortality in severe coronavirus disease 2019 patients with coagulopathy. J Thromb Haemost.

[7] Stone J, Hannge P, Albadawi H (2017). Deep vein thrombosis: pathogenesis, diagnosis, and medical management. Cardiovas Diagn Ther.

